# Comparing Prostate Imaging-Reporting and Data System Version 2 (PI-RADSv2) Category 1 and 2 Groups: Clinical Implication of Negative Multiparametric Magnetic Resonance Imaging

**DOI:** 10.1155/2020/2819701

**Published:** 2020-03-31

**Authors:** Jung Kwon Kim, Hak Jong Lee, Sung Il Hwang, Gheeyoung Choe, Sung Kyu Hong

**Affiliations:** ^1^Department of Urology, Seoul National University Bundang Hospital, Seongnam, Republic of Korea; ^2^Department of Radiology, Seoul National University Bundang Hospital, Seongnam, Republic of Korea; ^3^Department of Pathology, Seoul National University Bundang Hospital, Seongnam, Republic of Korea; ^4^Department of Urology, Seoul National University College of Medicine, Seoul, Republic of Korea

## Abstract

**Objectives:**

To evaluate the clinicopathological differences between Prostate Imaging-Reporting and Data System (PI-RADS) version 2 (v2) category 1 and 2 groups. *Materials and Methods*. We retrospectively reviewed our two institutional clinical databases: (1) transrectal ultrasound (TRUS)/magnetic resonance imaging (MRI) fusion biopsy cohort (*n* = 706) and (2) radical prostatectomy (RP) cohort (*n* = 1403). Subsequently, we performed comparative analyses between PI-RADSv2 category 1 and 2 groups. Clinically significant prostate cancer (csPCa) was defined as the presence of Gleason score (GS) ≥ 3 + 4 in a single biopsy core, and adverse pathology (AP) was defined as high-grade (primary Gleason pattern 4 or any pattern 5) and/or non-organ-confined disease (pT3/N1). We also performed multivariate logistic regression analyses for AP.

**Results:**

In the TRUS/MRI fusion biopsy cohort, no significant differences in detection rates of all cancer (18.2% vs. 29.0%, respectively, *P* = 0.730) or csPCa (9.1% vs. 9.9%, respectively, *P* = 0.692) were observed between PI-RADSv2 category 1 and 2 groups. There were no significant differences in pathologic outcomes including Gleason score (≥4 + 3, 21.2% vs. 29.9%, respectively, *P* = 0.420) or detection rate of AP (27.3% vs. 33.8%, respectively, *P* = 0.561) between the two groups in the RP cohort either. PI-RADSv2 category 1 or 2 had no significant association with AP, even in univariate analysis (*P* = 0.299).

**Conclusions:**

PI-RADSv2 categories 1 and 2 had similar performance to predict clinicopathological outcomes. Consequently, these two categories may be unified into a single category. Negative mpMRI does not guarantee the absence of AP, as with csPCa.

## 1. Introduction

In the past decade, there has been growing body of evidence that suggests a role of multiparametric magnetic resonance imaging (mpMRI) in the detection and management of prostate cancer (PCa). The latest European Association of Urology (EAU) guidelines on PCa strongly recommend mpMRI before repeat biopsy when clinical suspicion of PCa persists despite previous negative biopsies [1]. In 2016, the European Society of Urogenital Radiology (ESUR) released an updated guideline termed Prostate Imaging Reporting and Data System version 2 (PI-RADSv2) to ensure standardized and reliable radiologic criteria [2, 3]. In a previous meta-analysis, Woo et al. [4] have reported that PI-RADSv2 has high pooled sensitivity of 89% and specificity of 73%. However, they also demonstrated that there was significant variability in published results. Negative predictive value (NPV) to exclude clinically significant PCa (csPCa) was highly variable, ranging from 63% to 98% [5]. These variable results could be explained by differences in patient populations, reference standards, image acquisition techniques, image quality, interpretation criteria, reader experience, and interreader variability [4, 6].

Notably, in the majority of studies using PI-RADS, a category of ≥3 was considered as targetable lesion for biopsy to evaluate its performance in detecting PCa [1, 7–15]. Studies on PI-RADS category 1 and 2 lesions are lacking [11–14]. To the best of our knowledge, no studies have directly compared category 1 and 2 lesions. To further improve strategies, larger and longer-term follow-up data of these “negative” mpMRI patients would be essential. Accurate preoperative PCa prediction is essential for patient counseling as well as treatment approach. Accordingly, mpMRI plays an important role in predicting the final pathology at radical prostatectomy (RP). However, significant rate of mpMRI lesions does not always correspond to guided biopsy or RP specimen findings [16]. In addition, concordance rate between biopsy and RP Gleason score (GS) is only 35% to as high as 90% [17, 18]. Regarding this disconcordance, previous studies have demonstrated the association of PI-RADS with upgrading and extraprostatic extension (EPE) in RP pathology [19–21]. Thus, the aim of the present study was to evaluate pathologic outcomes after both biopsy and RP in patients with “negative” (PI-RADSv2 category ≤ 2) mpMRI. We also evaluated clinicopathological differences between PI-RADSv2 category 1 and 2 groups.

## 2. Methods

### 2.1. Ethics Statement

The Institutional Review Boards of the Seoul National University Bundang Hospital approved this study (Approval number: B-1706/402-115). As the present study was carried out retrospectively, written informed consent from patients was waived. Personal identifiers were completely removed and the data were analyzed anonymously. Our study was conducted according to the ethical standards recommended by the 1964 Declaration of Helsinki and its later amendments.

### 2.2. Study Cohort

#### 2.2.1. Transrectal Ultrasound (TRUS)/MRI Fusion Biopsy Cohort

We reviewed our institutional TRUS/MRI fusion biopsy database between September 2015 and March 2018 retrospectively. Accordingly, a total of 706 patients were included separately to the RP cohort.

#### 2.2.2. Radical Prostatectomy Cohort

We also reviewed clinical data of patients who underwent RP and preoperative 3-tesla (3 T) multiparametric prostate MRI (mpMRI) for clinically localized or locally advanced PCa between March 2008 and April 2018 at our institution retrospectively. A total of 1403 patients were finally included in this study after excluding 25 patients who underwent neoadjuvant treatment.

### 2.3. Preoperative MRI Protocol and Image Interpretation

All preoperative mpMRIs were performed after biopsy (usually 2 to 6 weeks later) using a 3 T system (Achieva Tx and Ingenia; Philips, the Netherlands) with a phase-array cardiac 6-channel coil without using the endorectal coil. mpMRI consisted of axial T2-weighted imaging (T2WI), T1/T2-weighted registered imaging (T1/T2RI), diffusion-weighted imaging (DWI) with corresponding apparent-diffusion coefficient (ADC) maps, and dynamic contrast enhanced (DCE). Detailed protocols were described in our previous reports [22–24]. All images were reviewed by two high-volume radiologists (H.J.L. and S.I.H.) who had >20 years of experience in interpreting prostate MRI using a Picture Archiving and Communication Systems workstation (PACS, INFINITT Technology, Seoul, Korea). mpMRI lesions were categorized through PI-RADSv2 [3]. Subsequently, all images from 2008 to early 2016 were rereviewed according to PI-RADSv2. Two radiologists were blinded to clinical characteristics including pathologic outcomes at the time of interpretation. However, they were aware that these patients had pathologically confirmed PCa.

### 2.4. TRUS/MRI Fusion Biopsy Protocol

From September 2015, two high-volume radiologists (S.I.H. and H.J.L.) in our institution started to conduct TRUS/MRI fusion biopsy [24]. 3 T mpMRI was performed before the biopsy in all patients. All mpMRI lesions were scored through PI-RADSv2, and images before early 2016 were also rereviewed according to PI-RADSv2. The fusion imaging technique (Volume Navigation; GE Healthcare, USA) with an electromagnetic field tracking system was used. Before the study, the axial MR images were uploaded from the PACS archive to the TRUS machine. After that, registration between the TRUS and MR images was performed to fuse both images correctly. In case of two index lesions in the same patient, the registrations were performed again for the subsequent lesion after the first biopsy. All TRUS-guided biopsies were performed with a Logiq E9 US machine (GE Healthcare, USA) equipped with a 5–9 MHz multifrequency endocavitary probe by the same uroradiologist who had conducted the image fusion [24]. An 18-gauge, 20 cm automatic cutting needle and an automated biopsy gun (ACECUT, TSK Laboratory, Japan) were used [24, 25].

Patients with index lesions of PI-RADSv2 category ≥ 3 were classified as the MRI-positive group, while patients with PI-RADSv2 category ≤ 2 as the MRI-negative group. Two cores of additional biopsy were performed per each index lesion under TRUS/MRI fusion. The maximal number of additional biopsy was four in a patient. Twelve cores of randomized systematic biopsy were followed. In the MRI-negative group, two cores of additional biopsy were conducted in the transition zone, followed by systematic biopsy. csPCa was defined as the presence of Gleason score (GS) ≥ 3 + 4 in a single biopsy core [8].

### 2.5. Data Acquisition and Definitions

RPs were conducted by several surgeons (S.E.L., S.S.B., S.L., and S.K.H.) using open, laparoscopic, or robotic modality. All pathological specimens were evaluated by a staff pathologist (G.C.) who had genitourinary expertise. Adverse pathology (AP) was defined as high-grade (primary Gleason pattern 4 or any pattern 5) and/or non-organ-confined disease (pT3/N1) [26]. The following variables were compared between categorical groups: age, body mass index (BMI), prebiopsy prostate-specific antigen (PSA) level, biopsy and pathologic GS, prostate volume on TRUS, PSA density (PSAD, serum PSA level/prostate volume) [27], and National Comprehensive Cancer Network (NCCN) criteria for prostate cancer risk assessment [28].

### 2.6. Statistical Analyses

Comparative analyses between PI-RADS categorical groups were conducted using a chi-squared test to evaluate the performance difference in predicting (1) TRUS/MRI fusion biopsy and (2) RP pathologic outcomes among different patient cohorts. In the subgroup analysis of PI-RADSv2 category 1 and 2 groups in the RP total cohort, the chi-squared test was used for categorical variables while independent *t*-test or Mann-Whitney *U* test was used for continuous variables to compare clinicopathological characteristics between the two groups. In addition, we performed receiver operating characteristic (ROC) curve analysis for AP prediction. We also performed logistic regression analyses to evaluate significant variables associated with AP. All statistical analyses were performed using IBM SPSS Statistics ver. 22.0 (Armonk, NY, USA), statistical package for R, ver. 2.13.2 (R Foundation for Statistical Computing (https://www.r-project.org/)). Statistical significance was considered when a two-sided *P* value was less than 0.05.

## 3. Results

### 3.1. TRUS/MRI Fusion Biopsy Outcomes

**Figure 1:**
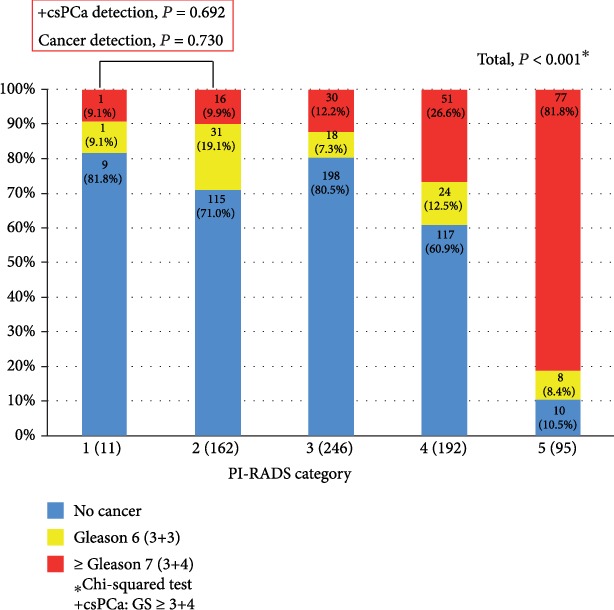
Prostate cancer detection stratified by Prostate Imaging-Reporting and Data System version 2 (PI-RADSv2) category in transrectal ultrasound (TRUS)/magnetic resonance imaging (MRI) fusion biopsy cohort.

Pathologic outcomes of TRUS/MRI fusion biopsy stratified by PI-RADSv2 categories are shown in Figure 1. There were significant (*P* < 0.001) differences in PCa/csPCa detection rates between groups stratified by PI-RADSv2 category. In contrast, there were no significant differences in these rates between PI-RADSv2 category 1 and 2 groups (all *P* > 0.05). Notably, patients with PI-RADSv2 category 1 or 2 had nonnegligible number of csPCa (9.1% and 9.9%, respectively).

### 3.2. RP Pathologic Outcomes in Total Cohort

**Figure 2:**
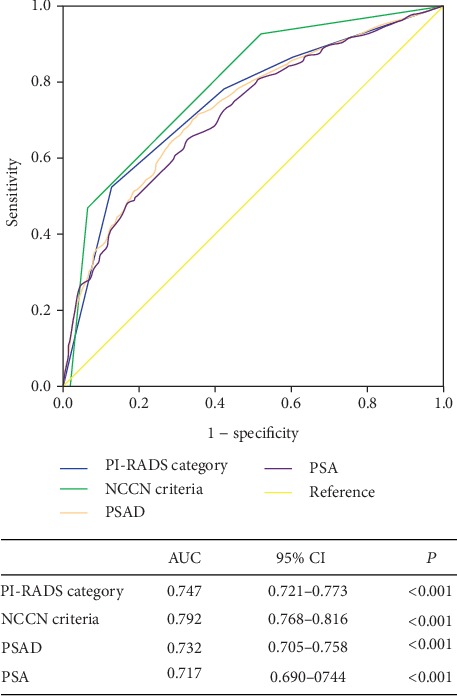
Receiver operating characteristic (ROC) curves for adverse pathology prediction in the radical prostatectomy (RP) cohort.

Among a total of 1403 patients, the rate of AP in RP specimen was significantly correlated with PI-RADS category resulting equal to 33.1% vs. 41.9% vs. 56.6% vs. 86.2% in the presence of PI-RADS category 1 or 2 vs. 3 vs. 4 vs. 5, respectively (*P* < 0.001, Table 1). ROC curve analysis for AP prediction showed an area under the curve (AUC) of 0.747 (95% confidence interval (CI): 0.721-0.773) for PI-RADS category (Figure 2).

### 3.3. Comparison of PI-RADS Categories 1 and 2 in the Subgroup of the RP Cohort

Results of comparative analyses of clinicopathological features between groups of PI-RADSv2 categories 1 (*n* = 33) and 2 (*n* = 311) are summarized in Table 2. There were significant differences in PSAD (categorical), prostate volume on TRUS, biopsy GS, and NCCN criteria (all *P* < 0.05) between the two groups. However, there were no significant differences in pathologic outcomes between the two groups (Table 3). In multivariate logistic regression analyses for evaluating variables associated with AP, diabetes, prebiopsy PSA, total prostate volume, and biopsy GS were found to be significant predictors of AP (all *P* < 0.05, Table 4). However, PI-RADSv2 category (1 vs. 2) had no significant association with AP, even in univariate analysis (*P* = 0.299; 95% CI: 0.664-3.779).

## 4. Discussion

Recently, there has been more emphasis on the role of mpMRI in diagnostic pathway for early PCa after the publication of two landmark studies [7, 8]. The PROMIS trial reported NPV of 89% for csPCa using 1.5-tesla mpMRI followed by both transrectal ultrasound (TRUS) biopsy and template prostate mapping (TPM) biopsy as a reference [7]. After that, the PRECISION trial compared a conventional diagnostic pathway (12-core TRUS-biopsy) and an MRI-directed pathway (MRI-targeted biopsy without systematic biopsy) and found that the MRI-targeted biopsy group showed superiority to conventional biopsy group in detection rate of both csPCa (38% vs. 22%; 95% confidence interval (CI): 4-20; *P* = 0.005) and clinically insignificant PCa (9% vs. 22%; 95% CI: -19 to -7; *P* < 0.001) [8].

Even with these practice-changing studies, obvious concern for this provision is still on debate. Based on PROMIS data, 11% of Gleason score (GS) ≥ 4 + 3 and 28% of GS ≥ 3 + 4 were missed on mpMRI [7]. They were even missed on TPM biopsy. In the PRECISION trial, 28% of patients in the MRI-targeted biopsy group did not undergo biopsy because they had a negative result on mpMRI [8]. In those patients, a significant number of csPCa might have been missed. Thus, systematic biopsies are still recommended for these patients although they have higher risk of complications along with economic burdens [9–11]. Several studies have reported a strong correlation between higher grade tumors and higher category of PI-RADS [12, 13, 19, 21]. Lim et al. [21] have demonstrated that higher PI-RADSv2 categories for GS 3 + 4 PCa detected at TRUS biopsy are associated with upgrading to GS 4 + 3 cancer and the presence of EPE after RP. They also reported that PI-RADSv2 category ≥ 3 was 100% sensitive for diagnosing GS 4 + 3 tumors. In the current study, we also found these strong correlations both in biopsy and RP pathologic outcomes (Figure 1 and Table 1). The rate of AP in RP specimen was significantly correlated with PI-RADS category: 33.1% vs. 41.9% vs. 56.6% vs. 86.2% in PI-RADS category 1 or 2 vs. 3 vs. 4 vs. 5, respectively (*P* < 0.001, Table 1).

Regarding PI-RADSv2 category 1 or 2, also known as “negative” mpMRI, several studies have reported a high NPV of mpMRI to exclude csPCa [7, 29, 30]. Subsequently, many authorities have recommended surveillance alone without biopsy for patients with PI-RADSv2 category 1 or 2 [31]. However, there is paucity of data concerning the intermediate and long-term follow-up and their correlation with RP pathologic outcomes [11]. In addition, according to a recent meta-analysis, there is no definitive conclusion about the NPV of mpMRI [4]. Voss et al. [10] have shown that over 28.9% of upgraded cases would have been missed, including GS ≥ 4 + 3 tumors. Even with the combination of PI-RADS category ≥ 3 and/or PSA density of ≥0.15, a significant number of men with intermediate-risk disease were misclassified. In the current study, we found that patients even with PI-RADSv2 categories 1 and 2 had a significant number of csPCa after TRUS/MRI fusion biopsy (17/173, 9.8% (Figure 1)). Among the patients who underwent RP with PI-RADS categories 1 and 2 in preoperative mpMRI, the rate of AP in RP specimen was as high as 33.1% (Table 1). With these findings, we can extrapolate to conclude that a significant number of patients with negative mpMRI may harbor csPCa and experience GS upgrading in their final pathology.

Current data on negative mpMRI are still lacking and studies regarding PI-RADS category 1 are scarce [9, 11–14, 20]. Bianchi et al. [20] have demonstrated the correlation between higher PI-RADS category and high probability of upgrading and upstaging after comparing PI-RADS category 2/3 vs. 4 vs. 5 and PI-RADS 2/3 versus 4/5. However, they did not include category 1 in their study. Until now, it remains unclear whether we can safely obviate biopsy, especially for PI-RADSv2 category 1 lesion. Notably, PI-RADSv2 categories 1 and 2 are not so different from each other in assessment. Category 1 lesion is defined as homogenous or normal signal intensity while category 2 lesion is defined as “indistinct” hypointense lesion on T2WI or ADC [3]. It would be essential to evaluate the performance of PI-RADS category 1 in predicting pathologic outcomes for further implementing surveillance strategies in this group of patients. In the current study, we found that there was no significant difference in PCa/csPCa detection rate between PI-RADSv2 category 1 and 2 groups after TRUS/MRI fusion biopsy (Figure 1). In the RP cohort, there were no significant differences in the rate of AP between the two groups (27.3% vs. 33.8%, *P* = 0.561, Table 3). Consequently, these results emphasize that performance improvement of mpMRI is needed for lesions classified as PI-RADSv2 categories 1 and 2. In addition, PI-RADSv2 category 1 should not be overlooked in the current mpMRI era.

In order to improve the performance of mpMRI in these groups, risk-assessment tools such as PSA, PSAD, Prostate Health Index, four kallikrein (4K) score, and Prostate Cancer gene 3 (PCA3) need to be combined [32–34]. In a recent prospective observational study, Lopci et al. [32] have reported that the use of ^68^Ga-labeled prostate-specific membrane antigen positron emission tomography/computerized tomography has significant performance in predicting csPCa among negative mpMRI patients.

The current study has some limitations. First, even with a large tertiary institution study, the retrospective study design was a crucial drawback. In addition, our study was based on a heterogeneous study cohort. For example, RP was conducted by several surgeons with several modalities and biopsy was conducted without standardized inclusion criteria. Finally, we did not conduct a rereview of pathologic slides. Accordingly, subsequent misclassification of some lesions might have affected outcomes. Further larger studies are warranted to validate and generalize our results.

## 5. Conclusions

The present study revealed that there were no significant differences between PI-RADSv2 category 1 and 2 groups in terms of the detection rate of csPCa. In addition, these groups had similar performance to predict pathologic outcomes at RP. Consequently, these two categories may be unified into a single category. However, these two categories defined as negative mpMRI do not guarantee the absence of AP, as with csPCa. Performance improvement of mpMRI is needed for these lesions. In addition, PI-RADSv2 category 1 should not be overlooked in the current mpMRI era.

## Figures and Tables

**Table 1:** Comparative analyses of pathologic outcomes according to PI-RADSv2 category in radical prostatectomy cohort.

*N* (%)	PI-RADS categories 1 and 2 (*N* = 344)	PI-RADS category 3 (*N* = 167)	PI-RADS category 4 (*N* = 385)	PI-RADS category 5 (*N* = 507)	*P*
Pathologic Gleason score					<0.001
6	30 (8.7)	15 (9.0)	11 (2.9)	1 (0.2)	
3 + 4	214 (62.2)	92 (55.1)	188 (48.8)	100 (19.7)	
4 + 3	85 (24.7)	49 (29.3)	152 (39.5)	233 (46.0)	
≥8	15 (4.4)	11 (6.6)	34 (8.8)	173 (34.1)	
Pathologic stage					<0.001
≤T2	305 (88.7)	137 (82.0)	293 (76.1)	217 (42.8)	
≥T3	39 (11.3)	30 (18.0)	92 (23.9)	290 (57.2)	
Adverse pathology, yes	114 (33.1)	70 (41.9)	218 (56.6)	437 (86.2)	<0.001

PI-RADSv2: Prostate Imaging-Reporting and Data System version 2.

**Table 2:** Baseline characteristics of PI-RADSv2 category 1 and 2 groups in subgroup of the radical prostatectomy cohort.

*N* (%) or mean ± SD	PI-RADSv2 category 1 (*N* = 33)	PI-RADSv2 category 2 (*N* = 311)	*P*
Age	65.6 ± 6.5	65.2 ± 7.3	0.778
BMI	24.1 ± 2.4	24.4 ± 2.7	0.562
Prebiopsy PSA	6.78 ± 3.8	8.86 ± 13.3	0.371
PSAD, continuous	0.17 ± 0.95	0.29 ± 0.64	0.258
PSAD, categorical			0.003
≤0.15	22 (66.7)	120 (38.6)	
>0.15	11 (33.3)	191 (61.4)	
Prostate volume	42.1 ± 10.7	35.9 ± 14.4	0.004
Biopsy Gleason score			0.002
6	25 (75.8)	135 (43.4)	
3 + 4	3 (9.1)	100 (32.2)	
4 + 3	4 (12.1)	53 (17.0)	
≥8	1 (3.0)	23 (7.4)	
NCCN criteria			0.002
Low	22 (66.7)	112 (36.0)	
Intermediate	9 (27.3)	164 (52.7)	
High	1 (3.0)	35 (11.3)	

BMI: body mass index; NCCN: National Comprehensive Cancer Network; PI-RADSv2: Prostate Imaging-Reporting and Data System version 2; PSA: prostate-specific antigen; PSAD: PSA density; SD: standard deviation.

**Table 3:** Comparative analysis results of pathologic outcomes between PI-RADSv2 category 1 and 2 groups in the radical prostatectomy cohort.

	PI-RADSv2 category 1 (*N* = 33)	PI-RADSv2 category 2 (*N* = 311)	*P*
Pathologic GS			0.420
≤3 + 4	26 (78.8%)	218 (70.1%)	
≥4 + 3	7 (21.2%)	93 (29.9%)	
Pathologic stage			1.000
≤T2	30 (90.0%)	275 (88.4%)	
≥T3	3 (9.1%)	36 (11.6%)	
Adverse pathology, yes	9 (27.3%)	105 (33.8%)	0.561

GS: Gleason score; PI-RADSv2: Prostate Imaging-Reporting and Data System version 2.

**Table 4:** Univariate and multivariate logistic regression analyses to detect adverse pathology in patients of PI-RADSv2 category 1 and 2 groups.

Variables	Univariate			Multivariate		
	OR	95% CI	*P*	OR	95% CI	*P*
Age	1.034	1.001-1.068	0.040	1.025	0.986-1.066	0.204
BMI	0.924	0.846-1.009	0.080	0.907	0.820-1.003	0.057
DM	2.204	1.206-4.027	0.010	2.639	1.281-5.436	0.008
HTN	1.052	0.670-1.654	0.825			
Prebiopsy PSA	1.044	1.010-1.078	0.010	1.050	1.006-1.095	0.025
Prostate volume	0.973	0.955-0.991	0.004	0.976	0.956-0.997	0.025
Biopsy Gleason score	7.240	4.377-11.977	<0.001	6.438	3.823-10.842	<0.001
NCCN criteria	4.836	3.119-7.501	<0.001	0.603	0.167-2.177	0.440
Clinical stage	1.329	0.890-1.985	0.165			
PI-RADSv2, 1 vs. 2	1.585	0.664-3.779	0.299			

BMI: body mass index; DM: diabetes mellitus; HTN: hypertension; NCCN: National Comprehensive Cancer Network; OR: odds ratio; PI-RADSv2: Prostate Imaging-Reporting and Data System version 2; PSA: prostate-specific antigen.

## Data Availability

The data that support the findings of this study are available from the corresponding author upon reasonable request.
